# What Morality and Religion have in Common with Health? Pedagogy of Religion in the Formation of Moral Competence

**DOI:** 10.1007/s10943-021-01279-6

**Published:** 2021-05-25

**Authors:** Zbigniew Marek, Anna Walulik

**Affiliations:** Jesuit University Ignatianum in Krakow, Kopernika 26, 31-501 Kraków, Poland

**Keywords:** Religion, Pedagogy of religion, Morality, Moral competence, Health

## Abstract

The aim of the article is to discuss how pedagogy of religion can contribute to the formation of moral competence and how this competence is in turn conducive to a new quality of life. Such analysis seems to be extremely important for modern educational theory. There are controversies concerning the role of morality in human life and its relationship with religion, and at the same time there is an increasing body of research that highlights the place and importance of religion in the formation of healthy individuals and societies. At the same time, the problem of the relationship between morality, religion and health is by no means new. The biblical writers already pointed out the connection between good and life, and between evil and death on the other hand. Both health and illness were taken to have their physical, inner, and spiritual dimensions. While Kulikova and Malchukova (2019) review the pedagogical and psychological subject literature, they lack references to the pedagogy of religion as a scientific discipline. This article aims to outline the Christian perspective on the formation of moral competence and its relationship with human health, understood as physical, mental, social and spiritual well-being. The understanding of this relationships is built on anthropological and transcendent foundations. Christian anthropological perspective means the acceptance of the so-called “personalistic norm.” Christian transcendent framework of moral education refers to the divine reality of the only God, the Creator and Redeemer of man. Moral competence is the result of moral education, which aims at engaging all human faculties—reason, emotions, and will—in discovering, accepting and internalizing values. One way of fostering moral competence based on the pedagogy of religion is to use the principles of the so-called pedagogy of accompaniment and testimony.

## Introduction

Discussion about the place and role of morality in human life triggers different emotions and opinions. One aspect that is controversial in this respect is religion. Questions are raised whether it is appropriate to resort to religion in matters of public life, whether it is a private or social issue. Is there a connection between morality and religion? And further, do morality and religion have anything to do with health? On the one hand, such questions make us aware of the natural need to discover the source and meaning of human existence and action, and, on the other, they express a sense of our own limitations and of the longing for a more solid foundation, giving a more complete sense of security and happiness.

These questions are not unrelated to the process of education. Research shows that the religious activity of young people is an effective antidote to experiencing emptiness and meaninglessness of life, as well as a protection against various risk behaviors. In addition, it is a response to problem situations experienced by young people Moreover, young people (Garbarino & Haslam, 2005) participating in the life of religious communities have a clearer sense of life and a more concrete philosophy of life than their peers who do not have such experiences (Markstrom, 1999). Young people, for whom their religiousness was important, were characterized by greater involvement in social activity compared to their irreligious peers (Youniss et al., 1999). Similar correlations were found in a study conducted on a group of 800 college students in Los Angeles. The results obtained reveal the existence of a positive correlation between the self-presentation as a religious person and the sense of life and social involvement (Furrow et al., 2004). These correlations can also be found in studies of adults of different ages (Walulik, 2009a; 2011). Other researchers focus on the individuals’ use of circumstances conducive to development in various areas of activity and their life management depending on the environmental conditions (Côté, 2000; Schwartz et al., 2005).

Some research indicates a direct relationship between the religious engagement of young adults and their identity development. It was discovered that people presenting the most mature form of identity according to James Marcia’s theory, i.e., identity achievement, sought both the information confirming and undermining their religious beliefs. These people at the moratorium level revealed a moderate level of religious doubt. In contrast, their peers in identity foreclosure were both more religious and less religiously doubtful in comparison with them. At the same time, they sought less contact with individuals who could pose a threat to their religious beliefs. Young adults in the status of identity diffusion did not engage in religious matters, harbored many doubts in religious matters, and avoided any consultation in this field. They also experienced various problems, while people in the status of identity achievement were well adapted (Duriez et al., 2008; Leak, 2009). Lawrence Kohlberg, the author of a major contemporary theory of moral development, highlighted the relationship between moral and religious development, stating that moral development is, to some extent, a condition for religious development (Kohlberg & Power, 1981). And further, James Fowler, (1981), the author of the concept of religious development, observed a strong relationship between moral thinking and religious thinking. It was a conclusion of his research, which involved 400 people in various stages of development.

Similar observations come to mind when we read the Bible. In the biblical narrative we can see a clear link between morality and health. In the Bible, life and good are synonymous (Deut 30:15), in contradistinction to death and evil, which are at the other extreme. The author of the Book of Sirach, numbered among wisdom books, simply states: “Health and strength are better than any gold, a robust body than untold wealth” (Sir 30: 15). What’s more, both health and illness concern both physical and internal dimensions. Moreover, some diseases such as leprosy or blindness have a symbolic meaning, and their understanding is discovered in man’s existential space, extending between good and evil, as well as between life and death. The categories of life and death are inextricably linked with the categories of health and disease. They are all factors that determine not only the biological and psychological, but also the spiritual and religious condition of a person. The issues of health and disease are naturally more complex than the binary consideration of life and death.

In the context of searching for relationships between religion, morality and health, it is worth to recall attempts to describe, not so much to define, what is behind the category of health undertaken by Józef Bogusz (1984). He refers to Aristotle, who recognizes that a person does not want to know what health is, but wants to be healthy. He also cites Arthur Schopenhauer’s famous aphorisms: “Health is certainly not everything, but without health everything is nothing” and “Probably a healthy beggar is happier than a sick king.” Thus, he implies that health is not only the absence of disease, but is a general human category (Gaweł, 2018). In a similar vein, health is defined by WHO (2018) as the well-being of a person in the physical, mental, social and spiritual dimensions. From the perspective of the pedagogy of religion, the spiritual well-being that a person may experience despite feeling certain deficiencies in other spheres is of particular importance.

The abovementioned categories lead to a reflection on the significance of the relationship between morality, religion and health for the processes of education aimed at achieving well-being. From the perspective of religious pedagogy, the fullness of human well-being is eternal life with God. Although—as stated by Kulikova and Malchukova (2019)—this problem is dealt with by both educators and psychologists, no such reflection was undertaken in the aspect of religious pedagogy, which is considered both a theological and pedagogical discipline.

The legitimacy of such deliberation is primarily due to the fact that this discipline focuses on the educational potential of various forms of religion and on religious education and socialization in the communities and in the society as a whole. It seems helpful to explore the new perspectives on the quality of human life. The following reflection engages the concepts of religion, pedagogy of religion, morality and competence. Their interpretation is narrowed to moral competences inspired by Christianity.

## The Conceptual Framework for Reflection on Morality and Religion

Pedagogy of religion develops theories of socialization and religious education. It provides the conceptual framework for the consideration of the role of morality and religion in education. Education takes place in various social environments: family, church, school, subcultures, peer groups, workplace and in volunteering. Researchers analyze the assumptions and practices of religious education both from the perspective of multidimensional interpersonal relations and from the perspective of religious teaching. In this sense, pedagogy of religion is considered to be a normative–empirical discipline that deals with the forms of affirmative and critical processing of religious content and human reflection on one’s own existence (Mette, 1994; Milerski, 2019; Boschki, 2017). The content for this deliberation is provided by religion.

Although the concept of religion is derived from different sources and is attributed different meanings, the authors agree that the essence of every religion understood subjectively is the recognition of human dependence on transcendence-God-deity. In objective terms of the subject, religion is identified with a set of beliefs, norms of conduct, ritual activities, and institutions. All the above explains and regulates the relationship of a person or larger communities to transcendence-God-deity (Mette 1994; Milerski, 2019; Marek, 2017; Boschki, 2017). Each religion also inspires a specific system of thought and action, supporting the person in overcoming her limitations and in her pursuit of the goal defined by Paul Tillich as “ultimate care” (Tillich, 1958). It should be noted that in our culture Christianity stands out among other religions as a religion of the Person, of the Triune God. This God in the person of Jesus Christ entered the history of mankind, respecting human freedom and dignity. Within history, He constantly works with love for human development, good and happiness called infinite life—immortality (Marek & Walulik, 2020). Christianity understood in this way significantly differs from all other religions, which, however, does not exclude the existence of contact points between them.

Another property of religions is that each one in its own way defines attitudes adopted in everyday life. They are identified with a constant responses to situations emerging in life and called moral behavior. Morality is the ability to perform concrete everyday actions directed toward good. Morality is characterized by accepted norms and rules, as well as specific habits that allow us to harmoniously function in social life. This faculty can be compared to an internal compass, which shows the path of moral conduct, and its main purpose is to internalize a specific world of values.

The concept of “competence,” similarly to the concept of religion, is also explained differently and is usually identified with such terms as: ability, fitness, skill, talent, property, disposition, responsibility, compliance, entitlement, the scope of someone’s knowledge. The reflection on moral competences in the context of religion requires that they be related to intercultural competences (Byram, 1997). These accounts of competences take them to be the dispositions of a person combining knowledge, skills, and attitudes, attained in the space of values and experience. They are a subjective category and belong to the educable dispositions. Their acquisition always occurs in a specific context and takes place in a specific set of situations that are typical of a particular environment. Hence, competences are dynamic and are subject to transformations throughout the entire human life. This assumption directly refers to the reflection on the harmonious functioning of man in personal and social life, i.e., on moral competence.

## The Anthropological Foundations of the Formation of Moral Competence

The understanding of moral competence depends on the adopted anthropological assumptions. Recognizing Christianity as the foundation for reflection on the relationship between morality and religion in education, we accept the so-called personalistic norm as an expression of Christian humanism. It assumes that the “phenomenon of man” has been fully revealed and explained by the Word–Jesus Christ. Humanism also emphasizes the unity of the physical and spiritual sphere of man. At the same time, it highlights man’s subjectivity, as well as his social nature, and the central position in the world, which is subordinate to him (Guzowski, 2007).

The harmonious functioning of a person in the world is the result of an interiorized world of values, which includes everything that she holds dear. There are both material and spiritual goods (knowledge, morality, law, religion, art). Making choices in the sphere of values requires such competences as the ability to express judgments and moral assessments and the willingness to accept the discovered norms of conduct (Mariański, 1989). Thus, morality becomes a kind of measure (criterion) for assessing behavior and a reference point in recognizing something as good or bad. Value is often equated not so much with a specific criterion determining whether something is valuable or contradicts it, but rather with the good itself. The notion of value is identified with the object of evaluation considered as good in itself (Scheler, 2009). Hence, it is said that values in educational processes constitute the so-called axiological category. Its enables the person to find the right orientation in what is important and valuable for her and for a larger community, and, consequently, what is desirable, what evokes positive feelings and reveals the purpose of life aspirations.

Representatives of the personalist trend emphasize that a person is such a good which cannot be treated as an object of use and thereby a means to an end. This goes hand in hand with the positive content of the personalistic norm, according to which a person is such a good that only love is a proper and fully valuable attitude toward her. In other words, the proper response to the dignity that characterizes a human person is affirmation through an attitude of respect, love and interpersonal solidarity. All this means that man should be perceived as a value that is significantly different from other innate or acquired values. The value of a person is associated with her entire being, not just her sex, aesthetic appearance, education, character traits, or other factors. The value of a person is quite specific and fundamental, and thus independent of any physical or psychological qualifications, of her external or internal possessions (Kupczak, 2000).

To these explanations, Christianity adds that man, who is the center of world life, is in his essence subordinate only to the personal God. This aspect indicates the unique dignity of man, which goes far beyond its natural status expressed in the ability to know, love, be free and to be the subject of rights and obligations. In the light of the truth about creation and incarnation, Christianity extends these human qualities, and clearly confirms that every human being has the highest value. Therefore, the human person can neither be mistaken for a thing, nor be bought or sold, nor exchanged for anyone or anything, regardless of her possessions, power or education. The specific aspect of the Christian view is that a person has been given the opportunity to establish relationship with God and become His partner in the development of creation, to participate in God’s wisdom and power and to be His image, in the equality of all people, and above all to participate in fulfilling the plan of salvation (Novak, 1997). What’s more, the awareness of human dignity and value seems to favor activities that foster the formation of moral competences. They refer to the spiritual–volitional–intellectual sphere (i.e., the sphere of consciousness, cognition, volition, and mental experience), and to the entire human being perceived in his spiritual and bodily integrity. This means that under no circumstances can the human person be appropriated either through manipulation or in the form of research experiments or as a tool to satisfy one’s desires or needs. Such awareness of her value demands that a person should always be considered as an end in itself and not as a means to an end (McGrath, et al., 2006).

## Transcendent Foundations of the Formation of Moral Competence

Another way in which pedagogy of religion can contribute to the development of a person and, consequently, to her moral growth, is by opening her to transcendence. Openness to transcendence manifests itself in human aspirations to know what is hidden and unknown. In this sense, transcendence means reality existing outside the world accessible to empirical and intellectual cognition, i.e., supernatural reality. This quest is pursued by world religions, including Christianity, which takes the world of transcendence to mean the world of God, the only God, the Creator and Redeemer of man. In simplified terms, it can be said that the effort put by individual religions in discovering supernatural reality focuses on the search for transcendence of the supernatural and divine in the immanence of the human world. Benedict XVI (2011) explains: “The religious dimension is an undeniable and irrepressible feature of man’s being and acting, the measure of the fulfillment of his destiny and of the building up of the community to which he belongs. Consequently, when the individual himself or those around him neglect or deny this fundamental dimension, imbalances and conflicts arise at all levels, both personal and interpersonal.”

The belief mentioned above demands corrections in our epistemic approach to the world and all it encompasses. The point is to accept religious explanations of reality in addition to empirical and intellectual knowledge. Such discovery of the surrounding reality poses new challenges to man. For in this form of assistance in the formation of moral competence, the motivation of the quest becomes vital. What is essential in the development of moral competence is both the kind of experience which is helpful in discovering one’s deeper needs and, above all, the desire to satisfy them. The motivation to search for truth and follow it in everyday life is also developed in the process. This awareness is also conducive to the development of moral attitudes, transforming them gradually from the initial heteronomic stage into the mature autonomous form.

Attaining such moral maturity requires vital competences, encompassing responsibility for oneself, for the other person, for the entire community, and, finally, for the surrounding world. Religion plays its role in such moral formation by unfolding the world of values and the reality of human destiny. In terms of the pedagogy of religion, one needs the ability to recall religious content when interpreting the experiences and events of human life. It is crucial to be convinced that openness to transcendence—in this case a personal God—is always directed toward man and his true good associated with human immortality—i.e., eternal life. Christianity equates the category of immortality, which is the possibility of achieving eternal life, with the category of happiness, which should not be confused with pleasure or satisfaction. Aristotle’s belief that people are not so much interested in what health is but rather desire to be healthy implies that health is a good that builds happiness. Admittedly, Aristotle considers health to be a so-called secondary good like friendship or wealth. However, if there are deficiencies in the sphere of secondary goods, they can also disturb the fullness of happiness (Świeżawski, 2000). In this sense, eternal life is an integral human well-being. Religion and religiously motivated morality provide a compelling inspiration to strive for it. The relationship between religion and morality is synergistic. The effect of the interaction of these factors in the process of religious education is moral competence.

## Moral Competence in the Perspective of Pedagogy of Religion

The existential, cultural, social and religious context of the relationship between morality, health and religion outlined above determines the goal of activities inspired by the pedagogy of religion. It is the change of a person’s life leading to the achievement of ultimate well-being—eternal life. This ultimate goal is realized through the achievement of intermediate goals, i.e., physical, mental, social and spiritual well-being. To reach this aim, pedagogy of religion takes into account the aspect of faith in line with personally accepted and socially established religious tradition (Bagrowicz, 2000). As a scientific discipline, pedagogy of religion integrates various research perspectives on religious education and socialization, thus generating praxeological knowledge, whereby religious education is seen as a task to be performed (Milerski, 2019). At the same time, pedagogy of religion refers to specific religious axiology, formulates value-based statements, and considers educational reality to be an area of realizing values. The axiological system associated with Christianity is also acceptable to non-believers, because it creates opportunities to learn the meaning of true tolerance and protects against the affirmation of tolerance beyond values and rationality.

The axiological plane grounded in the pedagogy of religion is instilled into the process of general (humanistic) education, understood as the whole of interactions occurring in the course of mutual relations between persons. Its important task is to equip the student with the skill of existential understanding, which manifests itself in an independent and perceptive interpretation of existence. This skill is fostered primarily through various cultural forms designed to help the student understand himself and the world in the context of his religion. It is crucial that the student learns how to interpret reality in the light of God’s revelation (Milerski, 2019). This means that we can talk about moral competence built on knowledge, skills and attitudes of a humanistic and religious nature acquired through experience and engagement with values.

According to the premises of the pedagogy of religion, competences are formed by knowledge that derives its content from both natural and supernatural (religious) sources. The former, natural, source is human intelligence and reason, as well as empirical research. The latter, supernatural, source is God’s revelation. It should be immediately mentioned that although both forms of knowledge complement rather than exclude each other, each of them has a different scope (John Paul, 1998). The main difference between them results from their distinctive sources and ranges.

In religious cognition, man recognizes the secrets of how the world functions by analyzing the sources God has given him (God’s revelation). However, this form of information only refers to reality that cannot be known through empirical experience or intellectual reasoning. Religious cognition is primarily related to existential questions about the origin and the purpose of human life, his destiny and the meaning of existence. The person finds answers to these questions in the certainty warranted by his faith. Therefore, it is the authority of God, not of human reason, that sanctions the discovered truth. This means that while knowledge appeals to man by force of rational argumentation, religion invokes the motivation of faith. At the same time, as in case of any interpretation, faith requires knowledge, which is the result of reflection on the sources of knowledge specific to a particular religion. This knowledge helps attain mature, and therefore “healthy,” religiosity and, consequently, morality (Walulik, 2009b).

Transferring these observations to the level of competences acquired by a person, it should be noted that they enable her to cope with society’s challenges and derive maximum benefits for her own development, which is assessed from a specific normative perspective. This does not mean, however, that a specific axiology can be imposed on development and permanently associated with it (Gössling, 2003). This observation reinforces the belief that pedagogy of religion adds a new quality to becoming responsible for one's own decisions in human endeavors. Competences acquired on the basis of natural and supernatural cognition foster enable the person to make moral judgments. Formation of moral competence is about helping to progress from unconscious incompetence to unconscious competence (Donati & Watts, 2005). This ability refers to the evaluation of norms and behaviors that man discovers and assesses with his conscience (Martin, 2010). We take the term “conscience” to mean the judgment formed by the person about the moral good or evil of their intended act, the performance of which becomes the source of internal approval or guilt and of self-perception as a good or bad human being (Ślipko, 2002). A properly formed conscience brings peace and a sense of security into human life (Marek, 2009). Thanks to it, the person builds the basis of her internal health by establishing mature relationships with herself, with other people and with Transcendence. At the same time, the very fact of establishing relationships does not yet guarantee the achievement of a specific level of internal health. It is necessary to know and respect the binding moral law.

This also applies to the good of health. In terms of morality, each person is responsible for their own health and that of others. In terms of fostering moral competence, Christianity offers additional assistance resulting from faith. Responsibility as a moral competence integrates the factors contributing to strengthening the well-being of a person both in the existential and ultimate dimension, which is eternal life with God.

## The Ways of Forming Moral Competence

Moral competence is the result of moral education, which is about engaging all spiritual faculties of man (reason, emotions and will) in discovering values and then accepting and appropriating them. One way to achieve it is suggested by the pedagogy of accompaniment rooted in *The Spiritual Exercises* of St. Ignatius Loyola (Marek, 2017). Its structure is based on four basic pillars: experience, reflection, action and evaluation, which is preceded by the realization that all human action—including education—takes place in a variety of life contexts. The context in which these processes take place plays an important role in developing moral competence based on this structure. This allows us to notice that all educational processes are conditioned by historical, socio-economic, and political factors, as well as by personal beliefs, predispositions, achievements or physical and spiritual deficiencies. Taking them into consideration in educational work helps the person in her search for truth, thus protecting her against the temptation of conformity or of radicalism. In addition, the fullest possible knowledge of social and personal determinants facilitates reflection on the experience gained and inspires to take specific actions. The knowledge of determinants of the concrete educational relationship helps build within it mutual trust, which is indispensable for the student’s development.

The formation of moral competence based on the principles of the pedagogy of accompaniment begins with experience. This concept is associated with an individual event that took place in the student's life. It can concern some aspect of human life: sensual, spiritual, external or internal. It always takes a particular, individual form, referring to the particular person. This fact does not preclude other people from having similar experiences. No experience can be “forced” either on oneself or on others. It can only be recognized. No experience is ultimate. Experiences provoke questions about the course and meaning of events. In addition to the notion of “experience,” pedagogy of religion uses the term “religious experience.” This kind of experience opens man to Transcendence (God) and to the ultimate sense of his own being. It helps to perceive and to respect the consequences of one’s relationships with God. In particular, it is about trust, love and faithfulness shown to God also in situations such as death that humanly speaking seem to be an obvious failure (Tillich, 1958). It also releases a new motivation in a person to be responsible and to engage in the service of good. Experience raises interest in a person and prompts to ask questions and make new assessments. It arouses hope for the transformation of one’s life, and also raises all kinds of anxieties. Accumulated experiences also trigger cognitive activities and facilitate the discovery and acceptance of specific values, including personal and moral values. These qualities of experience are enriched by the pedagogy of religion, which can interpret it according to not only natural, but also supernatural—religious—knowledge.

The next step in developing moral competence based on accompaniment is reflection on one’s experience. It is supposed to occasion the person’s internal development. It is accomplished by interpreting the meanings of experience gained, as well as by acquiring the ability to solve perceived problems and discovering new ways of transcending one’s own limitations. This kind of reflection helps interiorize, select and absolutize the values discovered in the analyzed experiences. In addition, it facilitates understanding of a particular experience. The condition of the fruitfulness of such reflection is its reliance on thorough and comprehensive knowledge of both natural and supernatural (or religious) character. It is also significant that the ability to objectively assess the explored reality protects man against indoctrination and manipulation (Marek, 2017). Pedagogy of accompaniment assumes that reflection provides the person who gathers experience with impulses to take action marked by responsibility for herself and for the community. So it is important to help the person recognize the world of values and to develop the attitudes, which will guide her in her future decisions. These activities should be considered as supporting her in creating a vision of her own life. In acquiring competence thus understood, an important role is played by the pedagogy of religion, which, resorting to supernatural cognition, creates a new perspective on life. Among the new motives, the hope of living with God for all eternity may take on significance.

Properly conducted reflection is the basis for assessing reality. This stage is the last in the structure of education based on the assumptions of the pedagogy of accompaniment. It is about assessment based on trust and respect undertaken together by the student and the teacher of everything that happened in the course of discovering experiences, reflecting on them and specific actions resulting from them. It is about paying attention to achievements and successes, as well as noticing shortcomings or even failures. This step is to contribute to the development of the ability to assess one’s own competence of responsible functioning in personal and social life. In addition, it allows one to discover the effects of one’s own intellectual and moral development.

The education based on the elements described above can be referred to thinking and acting based on the assumptions of the pedagogy of accompaniment (Marek, 2017) and testimony (Marek & Walulik, 2019). Both context and reflection as well as the evaluation of action serve to discover a new understanding of experience. This makes us aware that when talking about “understanding” one should keep in mind approaching the truth in such a way that it is not appropriated. That is to say that its light should penetrate the human interior and form his mentality and sensitivity, so that both the pupil and the educator could become “men of conscience” (Marek, 2009).

A man of conscience is characterized by harmony in all spheres of life of a person and society. The categories discussed in this article, such as religion, morality and health, all meet in the phenomenon of conscience. It enables us to recognize the reality popularly referred to as “clear conscience” as an important condition not only in the existential dimensions of health, but above all as a requisite for achieving the ultimate well-being—eternal life.

## Summary

In the reflection on the participation of the pedagogy of religion in the formation of moral competence, the question was raised about the legitimacy of invoking religion in public life, especially in education. We note that regardless of the adopted worldview, religion is not indifferent and can be significant in the formation of human life by inspiring the thinking and actions of the person and of entire communities, including educational systems. This affirmative answer to the question posed at the beginning arises from the fact that religion is an important element of culture and has impact on human life by answering questions about its meaning and purpose. Thus, it gives it a specific direction. This does not mean that without religion man will not find his direction. Rather, it implies that religion provides the frame of reference for moral thinking about ourselves and the world around us. It is also significant that it sensitizes to the need for an objective perception of reality in which a particular person has their rights and obligations not only toward herself, but also toward others, and this entails the need to care for harmonious social relations. It seems impossible to build these relations without moral competence at the appropriate level. This competence is a sign of the acquired and developed internal health of a person. It manifests itself, among others, in life optimism, openness to other people and willingness to help them.

Religion and morality can therefore be considered as factors strengthening the sense of the integrity of a person and harmony of individual spheres of life, and thus protecting health. Religion provides certain tools for achieving moral competence which, by forming an upright conscience, strengthens the person’s general psychosomatic well-being. At the same time, religion, without denying or diminishing the existential dimension of human life, emphasizes its transcendent dimension, which makes us aware of the temporary character of life on earth and the infinity of life with God. The discovery of this principle is facilitated by moral competence developed in the process of religious education. This competence, in turn, enables the person to adopt a mature view on the relationship between law and conscience.

The above presentation of the relationships between health, religion, morality and religious education does not pretend to be comprehensive and exhaustive. However, in view of the commonly used, functionalist definition of health, the text may provide a space for further enquiry into the scientific and social understanding of this category.
